# MicroRNA-218-5p inhibits cell growth and metastasis in cervical cancer via *LYN*/NF-κB signaling pathway

**DOI:** 10.1186/s12935-018-0673-1

**Published:** 2018-12-04

**Authors:** Yunsheng Xu, Qin He, Yiyi Lu, Fengxing Tao, Liang Zhao, Rongying Ou

**Affiliations:** 1 Laboratory for Advanced Interdisciplinary Research, Institutes of Translational Medicine, The First Affiliated Hospital of Wenzhou Medical University, Wenzhou, 325000 Zhejiang China; 2 Department of Dermatovenereology, The Seventh Affiliated Hospital, Sun Yat-sen University, No. 628 Zhenyuan Road, Guangming District, Shenzhen, 518107 Guangdong China; 3 Department of Dermatovenereology, The First Affiliated Hospital of Wenzhou Medical University, Wenzhou, 325000 Zhejiang China; 4 Department of Gynaecology and Obstetrics, The First Affiliated Hospital of Wenzhou Medical University, Nanbaixiang Street, Ouhai District, Wenzhou, 325000 Zhejiang China

**Keywords:** Cervical cancer, miR-218-5p, *LYN*, NF-κB signaling pathway

## Abstract

**Background:**

We are committed to investigate miR-218-5 effects on the progression of cervical cancer (CC) cell and find out the molecular mechanism.

**Methods:**

GSE9750 was obtained from GEO database and R Limma package was applied to filter out dysregulated genes. The pathways were enriched by GSEA software, ClusterProfiler and enrichplot packages to predict the function of DEGs. The binding sites of *LYN* were detected by miRanda and TargetScan. The miR2Disease database was used to find miRNAs related with CC. The expression of miR-218-5p and *LYN* were quantified by qRT-PCR and that of LYN protein was measured by western blot. The targeted relationships between miR-218-5p and *LYN* were verified by dual-luciferase reporter assay. Colony formation assays, wound healing, transwell invasion assay and flow cytometer analysis were performed to investigate the roles that miR-218-5p and *LYN* played in migration, invasion and death of cervical carcinoma. Xenografts established in nude mice were used to assess tumor growth in vivo.

**Results:**

The highly expressed mRNA *LYN* was selected by microarray analysis in GSE9750. NF-κB signaling pathway was enriched base on GSEA results. The expression of miR-218-5p was lower but *LYN* was higher in CC primary tumors compared with normal control. In addition, miR-218-5p could regulate the expression of *LYN* in HeLa cells negatively. Overexpression of *LYN* could promote cell migration and invasion, but inhibit cell death in vitro, and also promote tumor formation in vivo via activating NF-κB signaling pathway which could be reversed by miR-218-5p.

**Conclusions:**

MiR-218-5p suppressed the progression of CC via *LYN*/NF-κB signaling pathway.

## Background

As one of the most universal gynecological malignancies, cervical cancer (CC) was always accompanied with high mortality all around the world [1]. There are four main kinds of epithelial cervical tumors defined by WHO: squamous, adenocarcinoma, which the former takes up 70–80% of CC while the latter accounts for 10–15%, neuroendocrine tumors and other epithelial tumors including undifferentiated tumor [2]. Despite decades of progression, the prognosis for CC patients remains unsatisfactory, especially for those with advanced-stage tumors [3]. In seek of an earlier diagnosis and better prognosis, deeper understanding of genetic mechanisms about CC is necessary. The present study focused on cervical adenocarcinoma cells, and sought to unravel its mechanisms of progression and potential biomarkers.

MicroRNAs are a class of noncoding RNAs in a length of around 22 nucleotides, and play crucial roles in cell differentiation and in cancer proliferation [4]. Evidence showed that a variety of miRNAs expressed abnormally in CC tissues and were involved in tumorigenesis, progression and metastasis [5]. For instance, in the study of Zubillaga-Guerrero et al. [6], it was indicated that miR-16-1 were involved deeply in the cell cycle processes of CC, by suppressing CCNE1 gene, whose expression controlls the cells transition from G1 to S phase after transcription. Dong et al. [7] demonstrated that restoration of miR-218 restrained the growth of CC; furthermore, the overexpression of miR-218 had the potential to make CC cells sensitive to carboplatin. It’s reported by Kogo et al. [8] that the miR-218 ~ survivin axis suppressed CC progress through regulating oncogenicity, migration and invasion, and inhibiting survivin could improve outcome in CC. Another result revealed that HPV16 E6 suppressed miR-218 expression to promoted EMT expression and CC cell invasion, while miR-218 down-regulated EMT expression and inhibited invasion in CC through targeting SFMBT1 and DCUN1D1 [9]. Based on understanding of miR-218 as an important potential factor, we verified the correlation between miR-218-5p and CC, and explored a possible regulation axis underneath the surface.

MRNAs carry genetic information by encoding polypeptides or proteins which are involved in a series of biological processes such as differentiation, metabolism and neuronal signaling [10]. Diverse studies have uncovered certain mRNAs as key of mechanism in CC. For example, Xia et al. [11] found that *SIRT1* mRNA in PTX-resistant CC tissues expressed notably higher than that in PTX-sensitive ones. *SIRT1*, when knocked down, was also revealed to correlate with massive apoptosis in CC cells [12]. The expression level of another mRNA *Kir6.2*, was predicted to positively related to the KATP channel and to the inhibitory effect of glibenclamide, suggesting a potential strategy for CC [13]. In addition, Qin et al. [14] found that with the increasing of AITC concentration, the *Bax* mRNA expression was improved, while the *Bcl*-*2* mRNA expression went down in CC cells.

In order to screen possible mRNA targets of miR-218-5p in CC regulation, we combined existing finding with bioinformatic analysis, and discovered mRNA *LYN* as a possible target. *LYN* is a member of SRC family of protein tyrosine kinases, and a key factor in growth, differentiation and other essential cellular processes [15]. A few studies have indicated an unneglectable role of *LYN* in CC. For instance, Liu et al. [16] uncovered that *LYN* was remarkably higher expressed in CC tissues, inducing the growth of tumor. In another research, Bisht et al. [17] found that the timing, concentrations, and kinetics of the decreased protein levels of the signaling proteins were due to Raf-1, ERK1/2, and *LYN*. Nevertheless, research on the effect of *LYN* in CC and relevant mechanism were insufficient, and therefore pended. In the present research, we looked into the abnormal expression of *LYN* in CC, and predicted its potential role in miR-218-5p’s relationship with CC.

NF-kappa B (NF-κB), a transcription factor that intercedes antiapoptotic signals in several cancer cell types, could promote apoptosis in cancer cells [18, 19]. NF-κB has also been touched in CC related studies. For instance, Zhu et al. [20] revealed that the cross-talk between ER stress, autophagy, apoptosis, and the NF-κB pathways could regulate the CC cell mortality. Meanwhile, it was found to nicotine could stimulate HeLa cells migration and invasion in Wang et al. [21] study, possibly by activating PI3K/Akt and NF-κB pathways; on the contrary, LY294002, an inhibitor of PI3K, and the pyrrolidine dithiocarbamate (PDTC), a suppressor of NF-κB. For another, with the IKK signalosome through IκBK, Notch-1 activated NF-κB pathway in CC cells [22]. In our study, we investigated the correlation between NF-κB and CC, and its significance in between miR-218-5p, *LYN* and CC.

In the present study, we looked into the correlation of miRNA miR-218-5p, the mRNA *LYN* and the NF-κB pathway in CC cell lines, and sought to unravel the functioning pathway. Our studies have determined miR-218-5p’s downregulating effect on phenotypes of CC cells, and predicted a targeting relationship between miR-218-5p and *LYN*. NF-κB played significant role downstream of *LYN*, as appeared in our results. Together, our findings showed that miR-218-5p dose its anti-oncogene job through the *LYN*/NF-κB axis, which were also proofed later in vivo. The three factors in the co-functioning loop may bring new perspectives of potential biomarkers and clinical targets of CC.

## Methods

### Microarray analysis

The gene expression profile of GSE9750 was acquired from Gene Expression Omnibus (GEO), containing 33 primary tumors and 24 normal cervical epitheliums. R Limma package was applied to screen out the DEGs between primary tumors samples and normal samples. Data quality detect was conducted by box plot and quantile normalization was used for parallel experimental error elimination. Significant DEGs were searched by Empirical Bayes method, *P *< 0.05 and |logFC| > 1.

### KEGG pathway enrichment analyses

For deeper understanding of DEGs, enrichment of the functions and pathways were analyzed by GSEA v3.0 software. The Kyoto Encyclopedia of Genes and Genomes (KEGG) pathway gene set was utilized to perform pathway enrichment analysis. Default-weighted enrichment statistic was adapted to conduct the permutation for 1000 times with normalized *P *< 0.05 considered significantly enriched. Next, 7 of most highly up- and down- regulated results in GSEA reports were chosen to undergo graphics processing via “ggplot2” package. In addition, we employed R language “GSEABase” “ClusterProfiler” and “enrichplot” package to visualize the GSEA enrich result data.

### Tissue specimens

Following an institutional review board-approved protocol, primary CC specimens (n = 5) and matched normal adjacent cervical tissues (n = 5) were collected at The First Affiliated Hospital of Wenzhou Medical University, China, and snap-frozen in liquid nitrogen until RNA extraction. The clinicopathologic characteristics of CC patients were provided in Table 1. Written informed consent was received by each patient and our research was approved by the ethics committee of The First Affiliated Hospital of Wenzhou Medical University, Sun Yat-sen University.

**Table 1:** Clinicopathologic characteristics of cervical cancer patients

Characteristics	No. of samples (n = 5)
Age (years)	
< 50	3
≥ 50	2
FIGO stage	
I/IA/IB/IC	0
II/IIA/IIB	5
III/IIIA/IIIB/IV	0
Tumor size (cm)	
< 4	2
≥ 4	3
Grade	
1	0
2	5
3	0
HPV status	
16	5
18	0

### Immunohistochemistry (IHC)

The primary CC tissues and matched normal adjacent cervical tissues were diced into 5 mm × 5 mm sections. 4% paraformaldehyde dehydrated in graded ethanol was utilized to immobilize tumor tissues, which was then embedded in paraffin. Tissues sections were placed on poly l-lysine coated glass slides and then were de-paraffinized and hydrated. The tissues were incubated with anti-LYN antibody (Abcam, USA, ab33914) and Goat anti-rabbit IgG H&L (HRP) (Abcam, USA, ab6721). Antigen–antibody binding was visualized via application of 3,3′ diaminobenzidine (DAB) chromogen (K4368, Dako).

### Cell culture

The human normal cell line—End1/E6E7 and CC cell lines—HeLa, SiHa, C33A and HT-3 were obtained from the BeNa Culture Collection (Peking, China). Normal cells were incubated in Keratinocyte-Serum Free medium with 0.1 ng/ml human recombinant EGF, 0.05 mg/ml bovine pituitary extract, and additional calcium chloride 44.1 mg/l (final concentration 0.4 mM). And CC cell lines were maintained in Modified Eagle’s Medium with 10% FBS (Invitrogen-GIBCO, Grand Island, NY, USA). All the cells were cultured at 37 °C in 5% CO_2_ environment.

### Real-time quantitative analysis

Total RNA was extracted from tumor tissues and cancer cells by TaKaRa MiniBEST Universal RNA Extraction Kit (Ambion Inc., Austin, TX, USA). Then RNA was quantified by using NanoDrop. CDNA was synthesized through QuantiTect Reverse Transcription Kit (QIAGEN, FSQ-101, Japan). Kapa Biosystems Inc. (Boston, US) provided us with the real-time RCR kit. Gene transcription was quantified based on 2^−ΔΔCT^. Primer sequences were shown in Table 2.

**Table 2:** Primer sequences for qRT-PCR

Gene	Primer sequence
β-actin	F: 5′- AGCGAGCATCCCCCAAAGTT -3′ R: 5′- GGGCACGAAGGCTCATCATT -3′
LYN	F: 5′- TGTGAGAGATCCAACGTCCA -3′ R: 5′- AAACTGCCCTTGGCCATGTA -3′
MiR-218-5p	F: 5′- CGAGTGCATTTGTGCTTGATCTA -3′ R: 5′- TAATGGTCGAACGCCTAACGTC -3′
U6	F: 5′- CTCGCTTCGGCAGCACA -3′ R: 5′- AACGCTTCACGAATTTGCGT -3′
si-LYN	Sense: 5′**-** GCAUGGAGAAUGGUGGAAA -3′ Antisense: 5′- UUUCCACCAUUCUCCAUGC -3′
si-LYN control	Sense: 5′- GCAAGAGUAGGGUGUGAAA -3′ Antisense: 5′- UUUCACACCCUACUCUUGC -3′
MiR-218-5p mimics	5′- UUGUGCUUGAUCUAACCAUGU -3′
MiR-218-5p mimics control	5′- UCACAACCUCCUAGAAAGAGUAGA -3′
MiR-218-5p inhibitor	5′- AACACGAACUAGAUUGGUACA -3′
MiR-218-5p inhibitor control	5′- UUGUACUACACAAAAGUACUG -3′

### Cell transfection

The miR-218-5p mimics, miR-218-5p inhibitor, mimics NC, si-*LYN*, pCDNA3.1-*LYN* and NC plasmid vectors were synthesized by Shanghai GenePharma (China). HeLa cells at logarithmic phase were resuspended after digestion and incubated to 6-well plates at the density of 1 × 10^6^ cells/well. The confluence of cells reached 80–90% after incubation for 18–24 h. Then the culture medium without serum and antibiotics was added to the plate. Cell transfection was conducted through the Lipofectamine 2000 (Life Technologies) and continuous to be incubated for 48 h.

### Western blot analysis

Cells were washed twice using cooled PBS and were lysed using lysis buffer include PMSF. Extracts were incubated on ice for 20 min and spun down at 10,000 g for 20 min. Protein concentration was detected by BCA protein assay reagent (Pierce). Proteins were separated by 12% SDS-PAGE and transferred onto polyvinylidene difluoride (PVDF) membranes. Then, the membranes were firstly blocked in 5% defatted milk at room temperature for 1 h. And then incubated at 4 °C overnight with primary antibody—LYN (1:1000, ab137338), NF-κB p65 (0.5 µg/ml, ab16502), p-NF-κB p65 (1:5000, ab86299), IκBα (1:2000, ab7217), and p-IκBα (1:10,000, ab1233462), GAPDH (1:10,000, ab181602) diluted in 5% nonfat dry milk. Membranes were then washed and incubated for 2 h at room temperature with HRP-conjugated secondary antibodies IgG-HRP (1:10,000, ab6721). Finally, the membrane was captured using the ECL Reagent and visualized by western blotting analysis detection system (Thermo Fisher, China).

### Colony forming assay

HeLa cells were seeded in six-well plates at the density of 1 × 10^3^ with irrelevant sequence, miR-218-5p mimics, miR-218-5p inhibitor, si-*LYN*, pCDNA3.1-*LYN* or si-*LYN *+ miR-218-5p inhibitor at 37 °C with 5% CO_2_. Fourteen days later, the colonies were counted by a light microscope (BX50; Olympus, Tokyo, Japan) at a low-power field.

### Wound healing assay

Wound-healing assay was implemented to measure the migration ability of HeLa cells in vitro. HeLa cells were respectively transfected with NC, miR-218-5p mimics, miR-218-5p inhibitor, si-*LYN* + pCDNA3.1-*LYN*, si-*LYN* + miR-218-5p inhibitor. Cells were seeded in 6-well plates and cultivated to reach 90% confluence. A 2 mm cell scraper was used to conduct wounding. 0 h and 24 h later the HeLa cells that migrated across the baseline were observed by a light microscope.

### Transwell assay

Transwell assay was applied to examine invasion cells in a 24-well Transwell chamber with a layer of Matrigel (BD Biosciences, San Jose, CA). HeLa cells suspension containing 5 × 10^5^ cells was added to the upper chamber with DMEM. The lower chamber was added with DMEM with 10% FBS. After 24 h incubation at 37 °C with 5% CO_2_, cells on the interior of the inserts were removed with a cotton-tipped swab, and cells on the lower surface of the membrane were stained with gentian violet (Sigma-Aldrich; Merck KGaA) for 10 min at room temperature, rinsed with water, and finally dried and counted using a light microscope.

### Flow cytometry

Cells were transfected with one of the following: NC, miR-218 mimics, miR-218-5p inhibitor, si-LYN and pCDNA3.1-LYN or co-transfected with si-LYN and miR-218-5p inhibitor for 48 h. Apoptosis was observed by Annexin V-FITC Apoptosis Kit (BD Biosciences, CA, and USA) and differences in apoptosis was analyzed by FACScalibur (BD Biosciences). Normal cells, early-stage apoptosis cells, late-stage apoptosis cells and necrotic cells were counted by using CellQuest software.

### Luciferase assays

The targets of *LYN* were predicted using TargetScan (http://www.targetscan.org/) and miRanda (http://www.microrna.org/microrna/home.do), and miR-218-5p was entered. To validate that *LYN* was a target gene of miR-218-5p, wild-type (*LYN*-WT) or mutant (*LYN*-MUT) fragments of *LYN* 3′-untranslated region (3′UTR) containing the miR-218-5p binding site were amplified by Platinum Taq DNA polymerase (Life Technologies). Amplified PCR products were cloned in the pMIR-REPORT miRNA expression reporter vector (Life Technologies). The luciferase vector pMIR-REPORT (Firefly luciferase) and pRL-SV40 vector (Renilla luciferase, Promega) were used to establish recombine plasmid with *LYN*-WT and *LYN*-MUT. Different recombine plasmids were co-transfected into HeLa cells through Lipofectamine 2000 (Life Technologies) with miR-218-5p mimics or miR-218-5p control. Luciferase intensity was measured by Dual Luciferase Reporter Assay Kit (Promega) at 24 h post-transfection.

### In vivo tumor xenograft study

Six-week-old female BALB/c nude mice (Institute of Zoology Chinese Academy of Sciences, Shanghai, China) were harnessed for this experiment. The mice were randomly assigned to one of four groups: control (NC), transfected with si-*LYN* (si-*LYN*), transfected with pCDNA3.1-*LYN* (pCDNA3.1-*LYN*), and co-transfected with si-*LYN *+ miR-218-5p inhibitor (si-*LYN *+ miR-218-5p inhibitor). At first, 100 μl (1 × 10^7^ cells/ml) HeLa cells were, respectively, injected into 5 BALB/c mice in the right posterior flank subcutaneously. After injection, tumor was measured by width, length and height every 5 days when reaching 100 mm^3^. On the 20th day, tumor was weighed and mice were sacrificed for harvesting xenograft tissues. The study was approved by the ethics committee of The First Affiliated Hospital of Wenzhou Medical University.

### Statistical analysis

Experiments mentioned above were repeated at least thrice. Data of all assays are presented as the mean ± standard deviation. Statistical analysis was performed by GraphPad Prism. *P *< 0.05 was considered statistically significant.

## Results

### Identification of differentially expressed genes

**Fig. 1:**
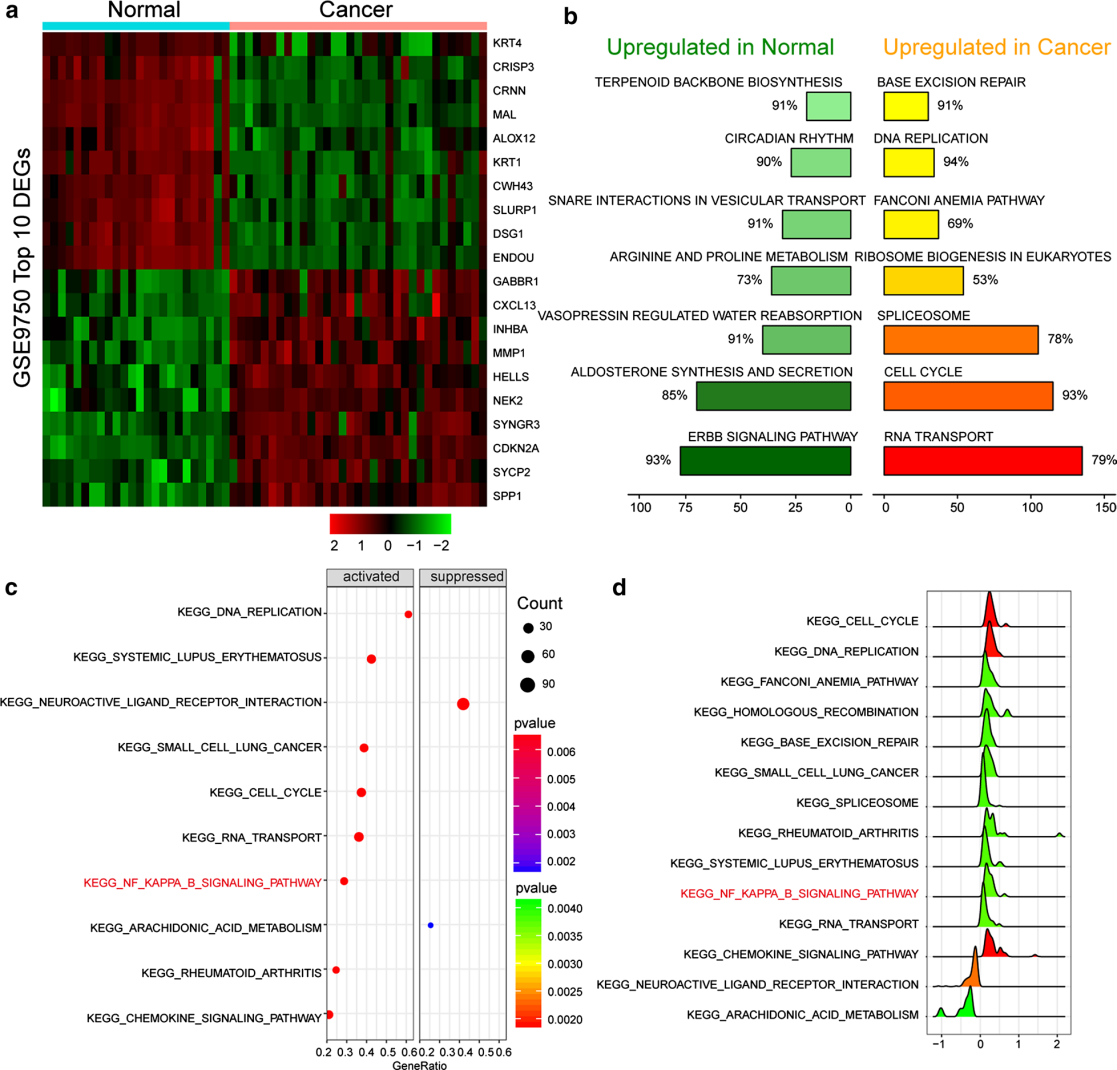
Function annotations for deregulated genes in cervical cancer. **a** The top 10 mostly up-regulated and down-regulated mRNAs in cervical cancer (CC). **b** Rankplot of seven most significantly enriched Biological pathway in normal and CC. **c** Dotplot of dysregulated KEGG pathways in CC. **d** Ridgeplot of dysregulated KEGG pathways in CC

After preprocessing and removing batch effects, the DEGs of GSE9750 was analyzed by Limma package. Using *P *< 0.05 and |logFC| > 1 as the cutoff criteria, a total of 1839 DEGs were identified, including 1020 upregulated genes and 819 downregulated genes in CC tissues compared to normal cervical tissues. The expression of top 10 mostly up- and down-regulated mRNAs are presented in red and green respectively on a heatmap (Fig. 1a).

### NF-κB signaling pathway was motivated in CC

**Fig. 2:**
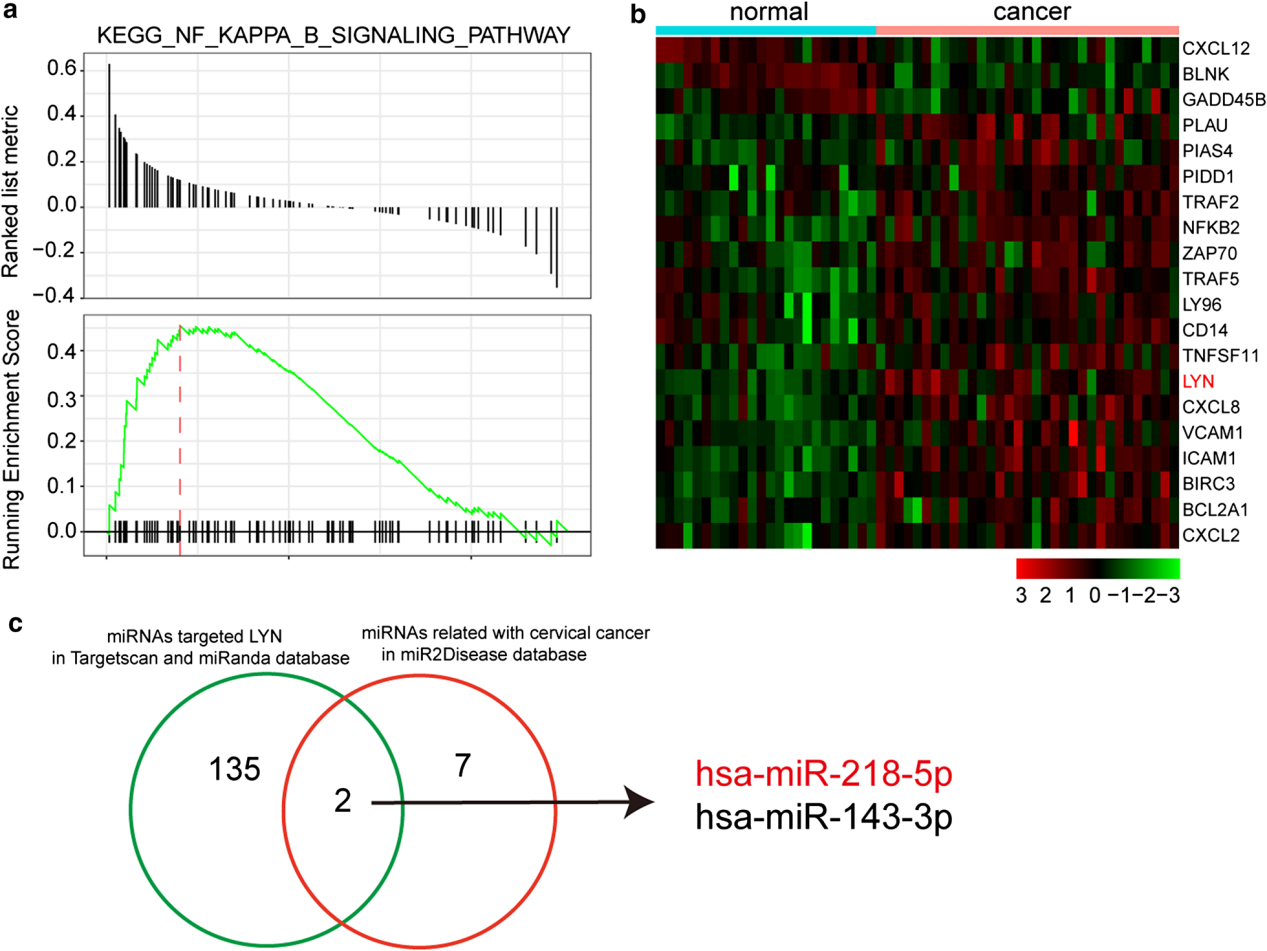
NF-κB signaling pathway was activated. **a** GSEAplot confirmed NF-κB signaling pathway was enriched (NES > 0) in CC. **b** Heatmap of DEGs expression which in NF-κB signaling pathway. **c** MiR-218-5p was chosen by overlapping miRNAs targeted *LYN* in Targetscan and miRanda database and miRNAs related with CC in miR2Disease database

GSEA report selected seven top scored pathways in CC (Fig. 1b). NF-κB signaling pathway was found activated in CC by dotplot and ridgeplot and further confirmed by GSEAplot (Figs. 1c, d, 2a). Furthermore, most mRNAs in NF-κB signaling pathway was highly expressed, including *LYN* (Fig. 2b). By overlapping miRNAs targeted *LYN* in Targetscan and miRanda database and miRNAs related with CC in miR2Disease database, there were two miRNAs, miR-218-5p and miR-143-3p (Fig. 2c).

### MiR-218-5p was downregulated and *LYN* was upregulated in CC

**Fig. 3:**
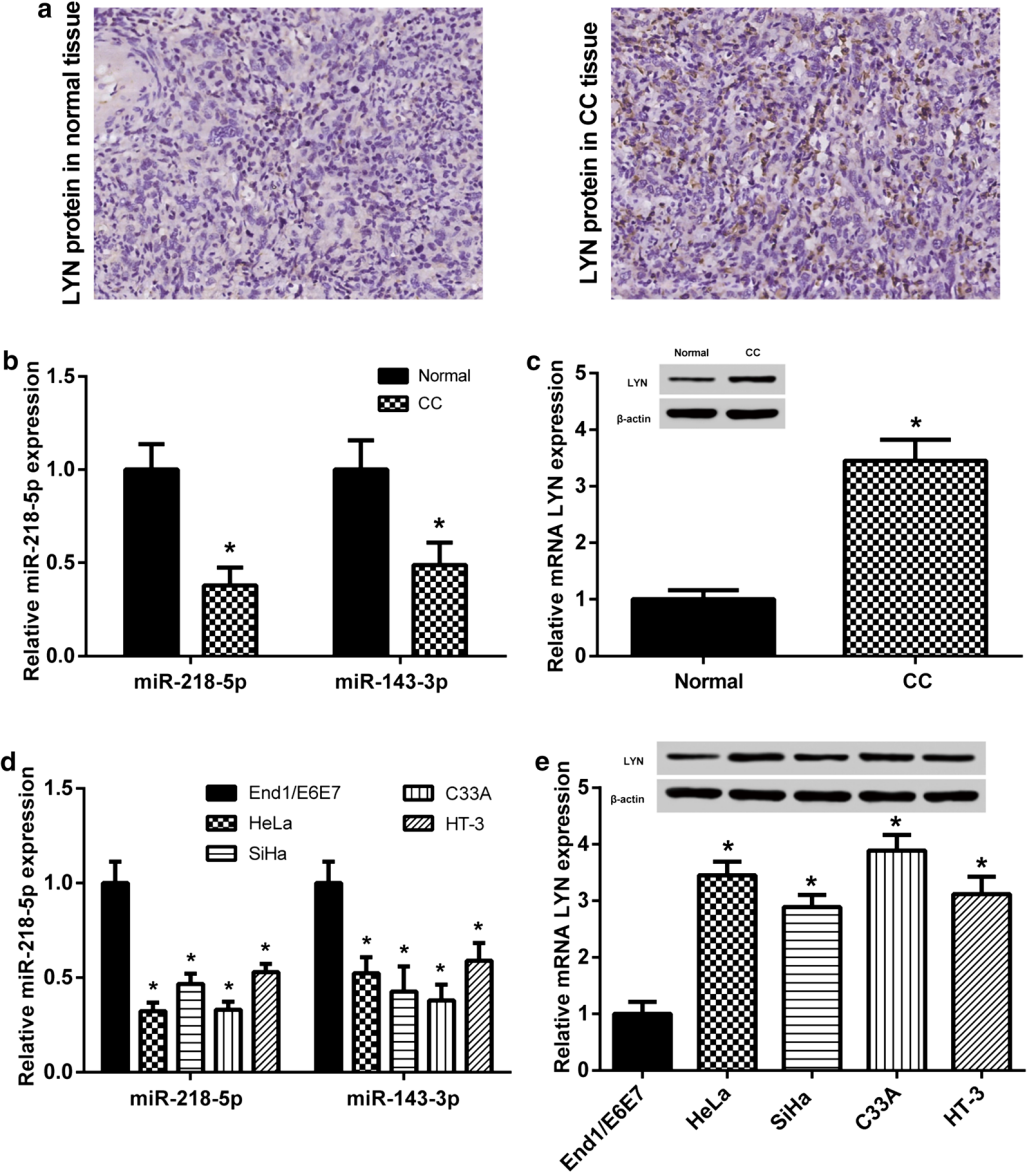
MiR-218-5p was downregulated and *LYN* was upregulated in CC. **a** *LYN* expression was higher in CC tissues than normal tissues by IHC. **b** MiR-218-5p and miR-143-3p expressions in CC were lower, *P < 0.05, compared with normal. **c** *LYN* expression was higher in CC tissues, *P < 0.05, compared with normal. **d** MiR-218_5p and miR-143-3p expressions in CC cell lines were lower, *P < 0.05, compared with the normal human cervical epithelial cell line END1/E6E7. **e** *LYN* expression was higher in CC cell lines, *P < 0.05, compared with the normal human cervical epithelial cell line END1/E6E7

According to the literatures, miR-218-5p and miR-143-3p were found downregulated in CC [9, 23]. MiR-218-5p and miR-143-3p were aberrantly decreased in CC tissues as well as cell lines HeLa, SiHa, C33A and HT-3 according to the consequences of qRT-PCR (Fig. 3b, d). Nevertheless, miR-143-3p expression levels in CC tissues and cells were relatively higher than miR-218-5p expression levels, and the changes of miR-218-5p and LYN expression were consistent in different CC cell lines. Hence, miR-218-5p was chosen for future study. The expression of *LYN* was opposite to miR-218-5p (Fig. 3a, c, e). The expression level of miR-218-5p was lowest in the HeLa cell line compared with that in other CC cell lines, whereas *LNY* expression level was relatively higher in HeLa cells. Therefore, HeLa cell line was chosen to perform the subsequent experiments.

### MiR-218-5p induced death of CC

**Fig. 4:**
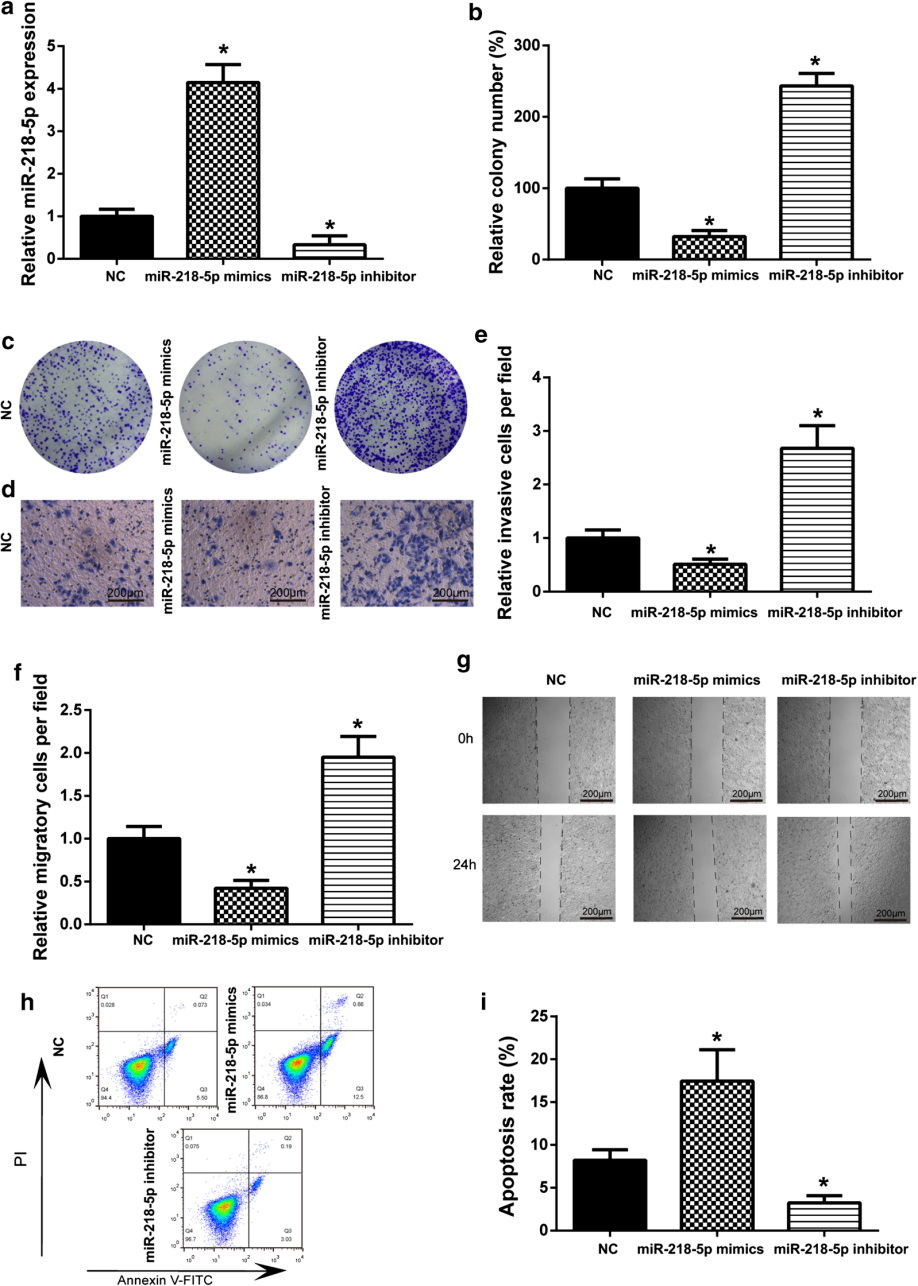
The effects of miR-218-5p on cervical cell proliferation, migration, invasion and death. **a** The expression of miR-218-5p was increased after transfected with miR-218-5p mimics, but miR-218-5p expression reduced after transfected with miR-218-5p inhibitor. *P < 0.05, compared with NC group. **b** HeLa cells transfected with miR-218-5p mimics showed an obviously decreased in cell proliferation, miR-218-5p inhibitor increased cell proliferation. *P < 0.05, compared with NC group. **c** Colony formation assay was used to detect HeLa cell proliferation. **d** Transwell assay was used to detect the invasion ability. **e** HeLa cells transfected with miR-218-5p mimics showed an obviously decreased in cell invasive, but transfected with miR-218-5p inhibitor showed significantly increasing. *P < 0.05, compared with NC group. **f** HeLa cells transfected with miR-218-5p mimics showed an obviously decreased in cell migration, but transfected with miR-218-5p inhibitor showed a significant increase. *P < 0.05, compared with NC group. **g** Wound healing assay was used to detect the migration ability. **h** Flow cytometry was used to detect the cell death. **i** MiR-218 mimics induced cell death than NC group; *P < 0.05

The expression of miR-218-5p was increased with miR-218-5p mimics, but reduced with miR-218-5p inhibitor (Fig. 4a). Colony formation assay displayed that miR-218-5p mimics had a remarkable inhibition on the cell proliferation (Fig. 4b, c). According to transwell assay and wound healing assay, miR-218-5p mimics led to suppression on the invasion and migration of HeLa cells (Fig. 4d–g). In addition, miR-218-5p overexpression significantly induced cell death in HeLa cells (Fig. 4h, i). The results were opposite when transfected with miR-218-5p inhibitor. In general, miR-218-5p could have a suppression of the growth of CC.

### MiR-218-5p targeted *LYN* mRNA in CC

**Fig. 5:**
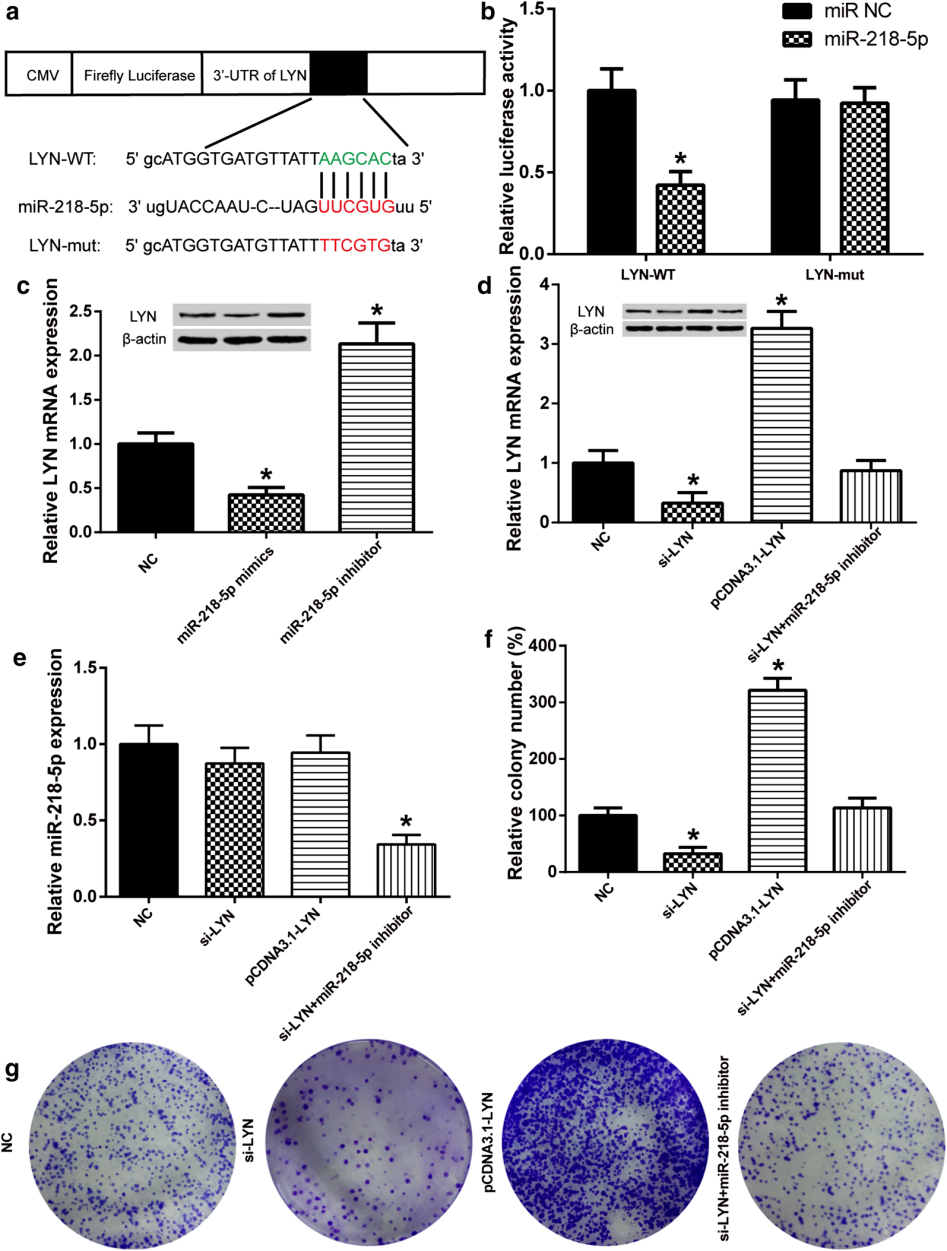
*LYN* was a direct target of miR-218-5p. **a** Schema of pMIR-REPORT vectors for the luciferase assay. **b** Relative luciferase activity in HeLa cells. *LYN*-WT + miR-218-5p showed a significant decrease. *P < 0.05, compared with other group. **c** The expressions of mRNA and protein of LYN were decrease transfected with miR-218-5p mimics. *P < 0.05, compared with the NC group. **d** The expression of mRNA and protein of LYN were decrease transfected with si-*LYN*. Co-transfected si-*LYN* + miR-218-5p inhibitor group had no change. *P < 0.05, compared with NC group. **e** The expression of miR-218-5p had no change when affected *LYN* expression. **f** *LYN* promoted cell proliferation in CC. **g** Colony formation assay was used to detect the effect of *LYN* for HeLa cell proliferation. *P < 0.05, compared with NC group

To confirm the targeting association, luciferase vectors containing *LYN*-WT or *LYN*-mut was constructed (Fig. 5a). Luciferase activity was much lower in wild type compared with the mutant group (Fig. 5b). This indicated that miR-218-5p targeted the 3′-UTR of *LYN*. The roles of miR-218-5p played in *LYN* expression in HeLa cells were investigated. *LYN* mRNA expression was obviously restrained by miR-218-5p mimics, and so as *LYN* protein. The result was opposite in miR-218-5p inhibitor group (Fig. 5c). The experimental results indicated that overexpression of miR-218-5p inhibited the expression of *LYN*. As shown in Fig. 5d, the expression of *LYN* was decreased by si-*LYN* but increased by pCDNA3.1-*LYN*, while the *LYN* expression remained unchanged when co-transfected with si-*LYN* and miR-218-5p. We also detected the effects of *LYN* on the expression of miR-218-5p but the consequences showed no effect (Fig. 5e). Overexpression of *LYN* increased cell proliferation compared with the NC group and *LYN* knockdown had the opposite effect (Fig. 5f, g). The effects of si-*LYN* on cell proliferation were reversed by miR-218-5p. In short, miR-218-5p had a negative regulation on *LYN* in HeLa cells.

### Overexpression of *LYN* promoted cell migration, invasion, and inhibited cell death

**Fig. 6:**
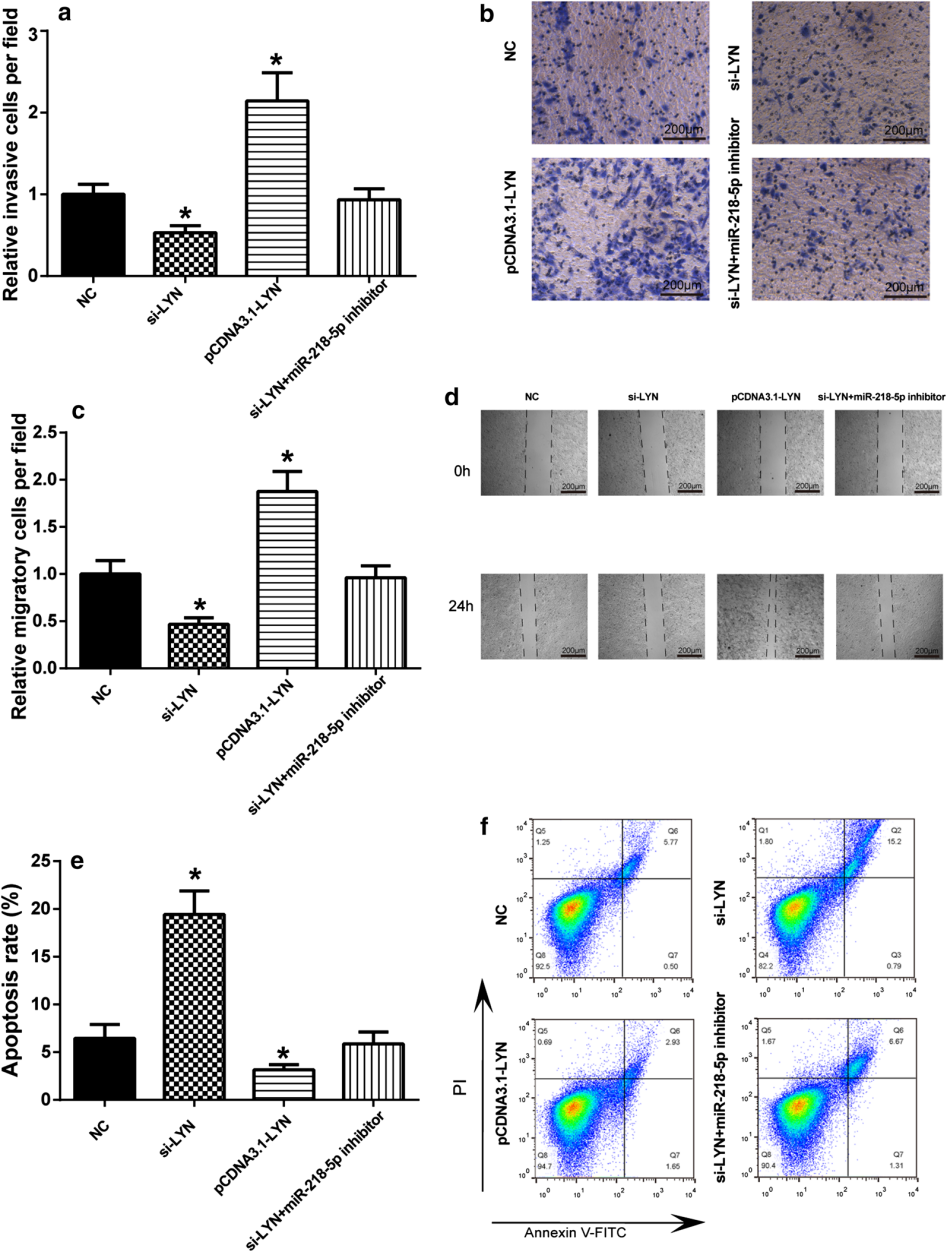
The effect of *LYN* on cell migration, invasion and death. **a** *LYN* promoted cell invasion in CC. **b** Matrigel invasion assay was used to detect the invasion ability of HeLa cells transfected with si-*LYN*, pCDNA3.1-*LYN* or si-*LYN *+ miR-218-5p inhibitor. *P < 0.05, compared with NC group. **c** *LYN* promoted cell migration in CC. **d** Wound healing assay was used to detect the migration ability of HeLa cells transfected with si-*LYN*, pCDNA3.1-*LYN* or si-*LYN *+ miR-218-5p inhibitor. *P < 0.05, compared with NC group. **e** LYN inhibited cell death in CC. **f** Flow cytometry was used to detect the death rate of HeLa cells transfected with si-*LYN*, pCDNA3.1-*LYN* or si-*LYN *+ miR-218-5p inhibitor. *P < 0.05, compared with NC group

Cell migration was detected by wound healing assay, cell invasion was observed by transwell assays and cell death was calculated by flow cytometry. When we transfected HeLa cells with pCDNA3.2-*LYN* to over-express *LYN*, the invasion ability of HeLa cells was increased remarkably (Fig. 6a, b). The similar results displayed in the cell migratory. Knockdown of *LYN* inhibited the migratory ability of HeLa cells (Fig. 6c, d). Flow cytometry showed that cell death increased significantly in si-*LYN* group (Fig. 6e, f). Hence, *LYN* attributed to migration and invasion of CC, and inhibited cell death while miR-218-5p reverse the effects of *LYN* overexpression.

### Overexpression of *LYN* activated NF-κB signaling pathway

**Fig. 7:**
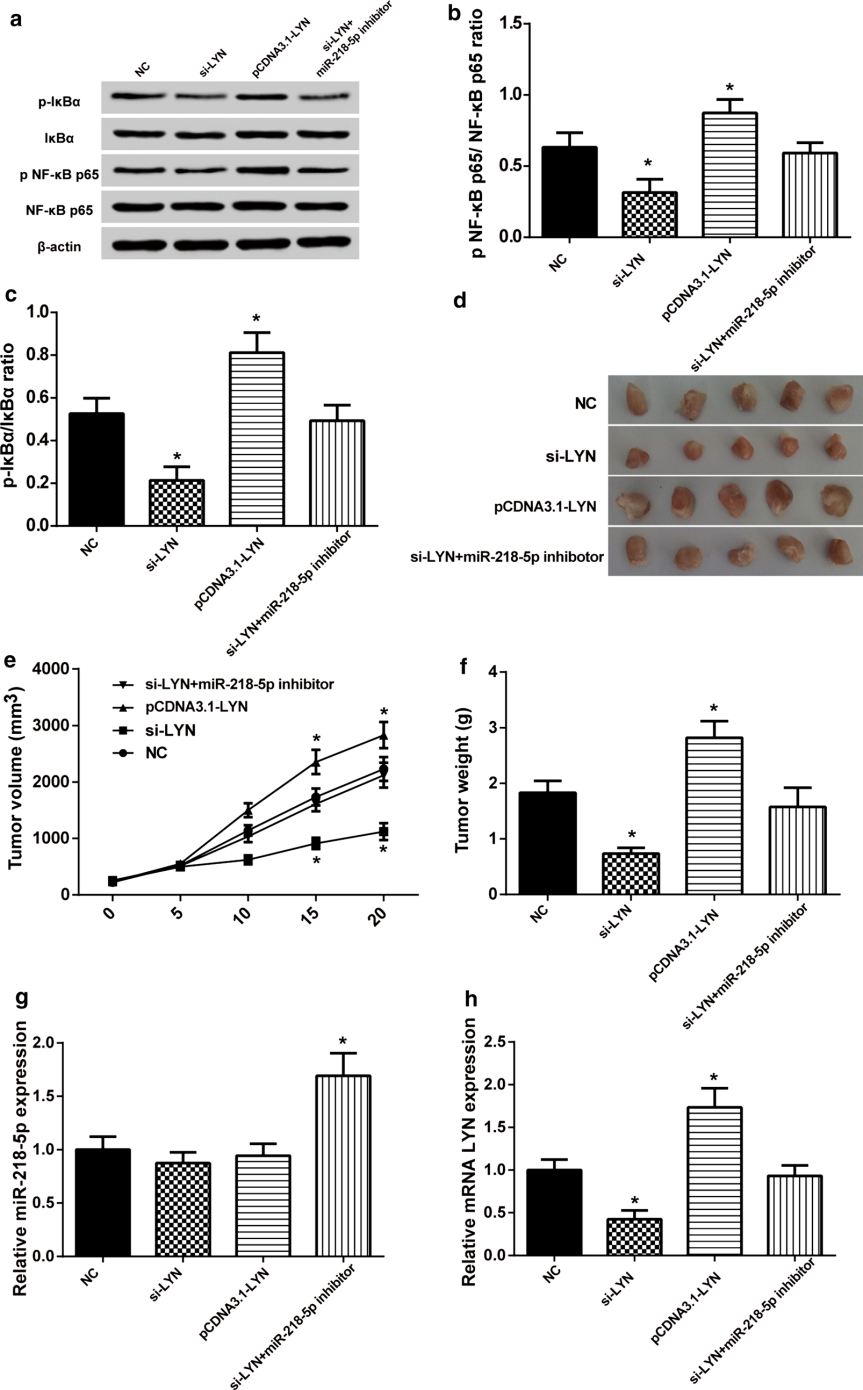
Overexpression *LYN* activated NF-κB signaling pathway and promoted tumor formation in vivo. **a** The protein level of NF-κB p65, p NF-κB p65, IκBα and p IκBα was detected by Western blot. **b** p NF-κB p65/NF-κB p65 ratio decreased in si-*LYN* group. *P < 0.05, compared with NC group. And p NF-κB p65/NF-κB p65 was unchanged in si-*LYN *+ miR-218-5p inhibitor group (**c**) p IκBα/IκBα ratio decreased when transfected with si-*LYN*. And p IκBα/IκBα was unchanged in si-*LYN *+ miR-218-5p inhibitor group. *P < 0.05, compared with NC group. **d** Effects of *LYN* on tumor growth of CC xenograft in nude mice. **e** The volume was smaller in si-*LYN* group and larger in pCDNA3.1-*LYN* group. *P < 0.05, compared with NC group. **f** The weight was lighter in si-*LYN* group and heavier in pCDNA3.1-*LYN* group. *P < 0.05, compared with NC group. **g** The expression of miR-218-5p was lower in si-*LYN *+ miR-218-5p inhibitor group in mice tumor tissues. *P < 0.05, compared with NC group. **h** The expression of *LYN* was lower in si-*LYN* group and was higher in pCDNA3.1-*LYN* group in mice tumor tissues. *P < 0.05, compared with NC group

To understand the molecular mechanism involved in NF-κB signaling pathway, the alterations in protein expression of NF-κB p65, p NF-κB p65, IκBα and p IκBα in HeLa cells were detected using Western blot analysis (Fig. 7a). Results showed that transfection with si-*LYN* decreased the ratio of p NF-κB p65/NF-κB p65 and p-IκBα/IκBα. On the contrary, when *LYN* was overexpressed, p NF-κB p65/NF-κB p65 and p IκBα/IκBα ratio significantly increased (Fig. 7b, c). MiR-218-5p reversed the effect of *LYN* on NF-κB signaling pathway. As a conclusion, we demonstrated that miR-218-5p down-regulated *LYN* expression and inactivated NF-κB signaling pathway.

### Overexpression of *LYN* increased tumorigenicity of CC cells in vivo

Compared with NC group, the average volume of tumor in si-*LYN* group was significantly smaller and weight was lighter than NC group. The results of pCDNA3.1-*LYN* group were obviously opposite, *P *< 0.05 (Fig. 7d–f). These data showed that miR-218-5p was under-expression only in si-*LYN* + miR-218-5p inhibitor group, and others group had no obviously change, *P *< 0.05 (Fig. 7g). The expression of *LYN* was lower in si-*LYN* group and higher in pCDNA3.1-*LYN* group, *P *< 0.05 (Fig. 7h). Therefore, these results of tumor xenograft study showed that *LYN* overexpression could increase the CC growth in vivo, and miR-218-5p could reverse the effects.

## Discussion

In the present study, differentially expressed genes (DEGs) were identified via microarray analysis of GSE9750. NF-κB signaling pathway was stimulated by GSEA. *LYN* was highlighted in highly expressed DEGs in NF-κB signaling pathway. The expression of miR-218-5p was low but *LYN* was high in cervical cancer (CC) primary tumors. Dual-luciferase reporting assay confirmed *LYN* was the target gene of miR-218-5p, and miR-218-5p negatively regulated the expression of *LYN*. MiR-218-5p, inhibiting *LYN*, had a negative control of cell migration and invasion via activating NF-κB signaling pathway. In addition, *LYN* promoted tumor formation in vivo, but miR-218-5p reversed the effects of *LYN*.

Nowadays plenty of bioinformatics methods were utilized to analyze the data. For example, differentially expressed genes (DEGs) and co-expression network analysis were wildly applied to seek modules of highly correlated genes. Through differential genes expression analysis of GSE9750 in our study, expression of 1839 mRNAs showed significant difference, in which 1020 were up-regulated and 819 were down-regulated. In the study of Xia et al. [24], two series from GEO datasets (GSE29570 and GSE89657) had been studied and performed an integrated analysis, exploring 61 key genes and ANLN was prominent among them. Zhao et al.’ [25] study indicated that p53 signaling was activated in CC by pathway enrichment analysis. Through bioinformatics analysis, two lncRNAs (lncRNA MIR100HG and lncRNA-AC024560.2) were identified, which provided broader perspective for preventing CC metastasis [26].

MicroRNAs (miRNAs) display dysregulated expression in human cancers and have a close association with carcinogenesis [27]. Our study observed the reduced expression of miR-218-5p in CC, and verified that miR-218-5p had the potential to induce cell death, and to inhibit the progression of CC in vitro. Similar regulating effects of miR-218 were also displayed in previous studies [28, 29]. Kogo et al. [8] also found that downregulated miR-218 was closely related to worse disease-free survival (DFS), overall survival (OS), and pelvic/aortic lymph node recurrence. Furthermore, Jiang et al. and Ben et al. proposed the expression of miR-218 was down-regulated by HPV type 16 E6 [9, 30]. Coincidentally, Martinez et al. and Li et al. had investigated the correlation between miR-218 and the presence of HPV infection in CC and the results revealed that the down-regulation of miR-218 was likely linked to the process of HPV-associated carcinogenesis in vivo [31, 32]. These findings suggested a novel therapy for CC based on miR-218-5p, especially for patients who had a low level of miR-218-5p [7].

In our research, *LYN* was a target gene of miR-218-5p and overexpressed in CC. *LYN* mRNA and protein caused cell proliferation, migration and invasion capability of breast cancer [33], chronic myelogenous leukemia [34], prostate cancer [35], colorectal cancer [36], oral cancer [37] and gastric cancer [38]. Liu et al. [16] revealed that overexpression of *LYN* was obviously involved in cancer differentiation and FIGO stages. NF-κB, a transcription factor, regulate cell proliferation and metastasis [39]. NF-κB signaling pathway was enriched by GSEA. In vitro experiment also demonstrated that up-regulated *LYN* expression activated NF-κB signaling pathway. However, several limitations still existed in our study. More disordered pathways in GSEA enrich results need deeper research. Other differentially expressed genes involved in NF-κB signaling pathway worth detailed study to gain deep understanding of mechanisms. Besides, the study falls on the small number of clinical samples, which, if larger, could bring more credibility to the expression difference of miR-218-5p and mRNA *LYN* between CC tissues and normal ones. For another, the correlations between miR-218/*LYN*/NF-B signaling pathway axis and HPV infection or pre-neoplastic lesions or with cancer progression were needed to further investigated in this study.

Our research has touched CC and its mechanism by exploring its correlation with miR-218-5p, and verified the correlation in vitro and in vivo. The study of miR-218-5p and the role of the *LYN*/NF-κB axis in it brings hints of new biomarkers to predict potential patients with higher risk of CC, and establishes possible targets of clinical therapies.

## Conclusions

In conclusion, our study unprecedentedly revealed that miR-218-5p restrained the activity of *LYN*, and inhibited the cell migration and invasion of CC. Moreover, knockdown of *LYN* had a negative effect on the growth of tumors in vivo. MiR-218-5p and *LYN* could serve as potential biomarkers for new targets for CC diagnosis and therapy. Therefore, through downregulating *LYN* and inactivating NF-κB signaling pathway, miR-218-5p obviously restrained CC progression.

## Abbreviations

CC: cervical cancer
DEGs: differentially expressed genes
DFS: disease-free survival
GEO: Gene Expression Omnibus
KEGG: Kyoto Encyclopedia of Genes and Genomes
OS: overall survival
PDTC: pyrrolidine dithiocarbamate
PVDF: polyvinylidene difluoride
